# Notes and News

**Published:** 1902-05-17

**Authors:** 


### NOTES AND NEWS.

On May 22nd the Lord Mayor will attend in State to open the new operating theatres at the London Hospital.

An Isolation Hospital fer sixteen beds has been opened at Chinley, in Derbyshire. The cost has been about £13,000. The architect is Mr. W. Bryden, of Buxton.

Thirty commissions as naval surgeons, and 12 commissions in the Indian Medical Service will be offered for examination, the first in June the latter in September next.

An open-air *fête* in aid of the Victoria Hospital for Children, Chelsea, will be held in July in the grounds of the Royal Hospital, Chelsea Embankment. The Prince and Princess of Wales have given their patronage to the entertainment.

Sir Peter Walker has offered, in commemoration of the King's Coronation, to present to the Derbyshire Royal Infirmary a complete installation of the Finsen Light, for the treatment of lupus. The estimated expenditure to carry out the proposal is £500.

The sixth Annual Medical, Surgical, and Hygienic Exhibition will be held at the Queen's Hall on May 20, 21, 22, and 23, from 1 p.m. to 10 p.m. Many novelties interesting to the medical profession will be shown, and there will be a concert each afternoon from 3 to 6, and in the evening from 7 to 10.

At the annual meeting of the National Sanatorium for Consumption, at Bournemouth, an unfortunate deficiency of £431, instead of a balance in hand as in the three preceding years, was shown on the accounts. This was attributed mainly to a falling off in donations, and to special expenses connected with efficiently carrying on the open air treatment of patients, now successfully adopted for some years past. Measures were considered for meeting the deficiency, and for obtaining increased funds for carrying out the proposed extension of the building (at a cost of about £1,200), and it was resolved that a further appeal for donations and subscriptions should be made to the public.

At Brisbane, Dr. Wray, the Health Officer, has died of the Plague.

The London City Mission announce that part of the year's work of the Association has been the reclamation of 1,519 drunkards.

Mr. William Turner will demonstrate surgical cases at his post-graduate lecture at the Westminster Hospital on May 20th at 4.30 p.m.

A convalescent home for co-operative trade societies has been opened at Gisland. Sixty co-operative trade societies contributed £16,000 for the purpose.

A case of plague has been reported on board the P. and O. steamer *Victoria* from Bombay. The patient is believed to have thrown himself overboard, and no other case has occurred. At Plymouth the passengers and crew were inspected, and the names of those landing both there and in London were taken.

At a recent meeting of the Royal Infirmary, Newcastle, the Mayor referring to the gift of a Finsen Light Apparatus from the Danish Consul in Newcastle, said that he thought it very unsatisfactory that no use should be made of it until the opening of the new infirmary, which meant a delay of two or three years.

At the annual competition held by St. John's Ambulance Brigade for the representatives of the railway companies the Metropolitan team gained the railway challenge shield. Sir John Furley, in his address to the men, pointed out that the St. John's Ambulance Association had sent out over 2,000 men to the war to aid the wounded.

The Lord Mayor will hold a meeting at the Mansion House on Monday, June 9, in support of King Edward's Hospital Fund for London. The Lord Mayor acquainted the King with the fact, and received in reply a letter from Sir Francis Knollys, saying that no scheme of a benevolent description in connection with the commemoration of the Coronation more deeply appeals to His Majesty than that for augmenting the Hospital Fund, and that the meeting has his warmest sympathy.

Mr. J. K. Caird has intimated his desire to found in connection with the Dundee Royal Infirmary a cancer hospital, and to endow research in cancer for five years. The design which has been submitted to the directors shows two pavilions connected by ventilated passages to a central block in which is placed the entrances, staircase, elevator, day rooms, and operation theatre. There will be room for over 100 beds, and these may be used for ordinary purposes provided sufficient accommodation be always kept for cancer cases. The estimated cost of the building is £18,500, and £5,000 will be spent upon cancer research. Mr. Caird presented the Royal Infirmary some years ago with a hospital for women, in which the gynæcological and maternity departments are now located, and a nurses' home in connection with them, at a cost of over £8,000. Details of the plans will be given in a forthcoming number.

